# Trends in the Incidence of Bronchopulmonary Dysplasia after the Introduction of Neurally Adjusted Ventilatory Assist (NAVA)

**DOI:** 10.3390/children11010113

**Published:** 2024-01-17

**Authors:** Kashish Mehra, Mitchell Kresch

**Affiliations:** Division of Neonatal-Perinatal Medicine, Penn State Health Children’s Hospital, Hershey, PA 17033, USA; mkresch@pennstatehealth.psu.edu

**Keywords:** VLBW, NAVA, BPD

## Abstract

Objective: This study investigates the difference in the rates of bronchopulmonary dysplasia in very low birth weight infants before and after the introduction of neurally adjusted ventilatory assist (NAVA). Study Design: A retrospective cohort study comparing rates of Bronchopulmonary dysplasia (BPD) before and after implementation of NAVA. Eligibility criteria included all very low birth weight VLBW neonates needing ventilation. For analysis, each cohort was divided into three subgroups based on gestational age. Changes in the rate of BPD, length of stay, tracheostomy rates, invasive ventilator days, and home oxygen therapy were compared. Results: There were no differences in the incidence of BPD in neonates at 23–25 6/7 weeks’ and 29–32 weeks’ gestation between the two cohorts. A higher incidence of BPD was seen in the 26–28 5/7 weeks’ gestation NAVA subgroup compared to controls (86% vs. 68%, *p* = 0.05). No significant difference was found for ventilator days, but infants in the 26–28 6/7 subgroup in the NAVA cohort had a longer length of stay (98 ± 34 days vs. 82 ± 24 days, *p* = 0.02), a higher percentage discharged on home oxygen therapy (45% vs. 18%, respectively, *p* = 0.006), and higher tracheostomy rates (3/36 vs. 0/60, *p* = 0.02), compared to the control group. Conclusions: The NAVA mode was not associated with a reduction in BPD when compared to other modes of ventilation. Unexpected increases were seen in BPD rates, home oxygen therapy rates, tracheostomy rates, and the length of stay in the NAVA subgroup born at 26–28 6/7 weeks’ gestation.

## 1. Introduction

Advancements in neonatal care over the years have led to increased survival in extremely premature infants, but there are 10,000–15,000 new cases of bronchopulmonary dysplasia (BPD) annually in the US alone [1,2]. The incidence of BPD in very low birth weight (VLBW) infants has remained stable at approximately 40% over the last few decades [3,4,5], and it remains the most common morbidity of preterm infants. BPD is a chronic illness that affects infants even after discharge, with problems such as an increased risk of re-hospitalization in the first year after birth, reactive lung disease [6], and poor neurodevelopmental outcomes [7].

The pathophysiology of BPD is known to be multifactorial in origin. One of the pathways stressed frequently is an imbalance between pro- and anti-inflammatory factors [8,9,10,11,12]. The majority of preterm infants require invasive or non-invasive positive pressure ventilation. Inflation of the lung with large tidal volumes has been shown to cause the disruption of structural elements in the lungs, which promotes the inflammatory cascade with the release of cytokines and interleukins, leading to fibrosis within the extracellular matrix [13,14,15,16,17]. Ventilator-induced lung injury (VILI) is known to contribute to the development of BPD [18]. Extended periods of positive pressure ventilation can lead to lung injury [19], secondary to volutrauma [14], which can presumably lead to bronchopulmonary dysplasia (BPD). Various lung protective and preventative treatment strategies have been tried but only a few have been shown to decrease the risk of BPD, such as non-invasive support (e.g., nasal CPAP), volume targeted ventilation [20], and caffeine [21] and Vitamin A administration [22]. We still lack effective strategies to prevent neonatal lung injuries and BPD.

Patient–ventilator synchrony is one of the essential ventilator parameters that needs to be monitored to prevent and reduce ventilator-induced lung injury. Asynchrony in neonates is mainly due to leaks around the endotracheal tube, a high respiratory rate, and a high variability in breathing patterns [23]. The synchronization of respiratory effort with ventilator inflation reduces asynchrony and is associated with improved oxygenation and ventilation [24]. Patient–ventilator asynchrony has been shown to be associated with worse outcomes in both pediatric and adult patients [23,24].

The best approach for physiological patient–ventilator synchrony would be to use the neuronal firing in the brainstem to synchronize the patient’s respiratory effort with the ventilator. Such technology is not available at this time. However, neurally adjusted ventilatory assist (NAVA) is a relatively new mode of ventilation that uses the electrical activity of the diaphragm (also called Edi signals) as a trigger to synchronize a patient’s respiratory efforts with the ventilator. There can be significant delays in pneumatic triggers in conventional ventilators using pressure or flow sensors [25], which can lead to asynchrony. Since neuronal firing (used as a trigger in NAVA) always precedes muscle contraction and changes in flow and pressure in the respiratory tract, the NAVA mode of ventilation shows better patient–ventilator synchrony [26,27,28,29,30]. NAVA has been successfully used in very low birth weight infants as small as 640 g [23,31]. Non-invasive NAVA has also found to be effective in settings of hypoxemic respiratory failure [32] and remains effective even with large leaks around the airway interface.

The primary aim of this study was to determine if there is a difference in the incidence of BPD after the introduction of the NAVA mode of ventilation. Secondary outcomes measured in the study were ventilator days, the length of the stay, and rates of home oxygen therapy at discharge.

## 2. Methods

### 2.1. Study Design and Groups

This is a retrospective, cohort study performed at the Penn State Health Children’s Hospital NICU (Level 4 NICU). The study period included patients from a time period between January 2011 and December 2017. The NAVA mode of ventilation was introduced in the NICU in January 2015. The control group included 4 years of historical controls from January 2011 to December 2014. The NAVA group included patients from January 2015 to December 2017. There were 109 patients in the control group and 100 patients in the NAVA group. Patients in each group were further subdivided into subgroups based on gestational age, i.e., 23–25 6/7 weeks’, 26–28 6/7 weeks’ and 29–32 weeks’ gestational age.

### 2.2. Use of NAVA

The NAVA mode was used with a SERVO-i™ ventilator. The NAVA mode uses diaphragmatic electric signals, or Edi, to synchronize patient effort with the ventilator. These electric signals are captured and monitored via miniaturized electrodes placed on a specific nasogastric feeding tube that can be advanced into the stomach, with the electrodes positioned at the level of the diaphragm. The product of Edi signal and NAVA level (decided by the provider) converts this electric signal into a proportional pressure assist delivered to the patient. There were no policies/guidelines regarding choosing an initial NAVA level; instead, it was based on the patient’s clinical status and the provider’s clinical judgment. NAVA levels were adjusted based on data from the Edi signals (normal Edi signal: 5–15 µV). Back-up NAVA ventilator settings were set by the physician based on their clinical judgment. The NAVA mode is available for both invasive and non-invasive ventilation.

The Servo-I ventilator collects data regarding the amount of time a patient is apneic and uses the back up pressure-controlled mode instead of NAVA in real time, but this information was not available for the current retrospective study because such information is not routinely documented in electronic medical records. The decision to administer the NAVA mode vs. a conventional ventilator/high frequency mode was solely based on physician preference, with no preset criteria.

Control group patients were on high frequency jet or oscillatory ventilators, synchronized pressure-limited volume-targeted conventional modes of ventilation, and/or non-invasive support with nasal continuous positive airway pressure, and high-flow nasal cannula greater than 2 LPM. The NAVA group patients were nonexclusive, as these patients were supported with the NAVA mode, as well as the other modes of ventilation previously mentioned.

### 2.3. Inclusion and Exclusion Criteria

Patients were eligible for this study if their birth weight was less than 1500 g and/or gestational age less than 30 weeks’. Patients were included in the study if they were inborn or outborn (transferred within 24 h of birth) and needed invasive or noninvasive ventilator support for any amount of time. Positive pressure ventilation included any type of ventilator support, continuous positive airway pressure (CPAP), or high flow nasal cannula greater than 2 LPM for any duration of time. Data collection was stopped when the patients were switched to a home ventilator after tracheostomy or weaned off all respiratory support. Neonates were excluded if they had a congenital heart disease that required surgery except Patent Ductus Arteriosus (PDA) ligation, had congenital airway or pulmonary malformations, or if they had any lethal chromosomal anomalies.

### 2.4. Measured Outcomes

BPD was defined in our study as per National Institute of Child Health and Human Development (NICHD) guidelines, i.e., oxygen requirement at 28 days of age and another evaluation at 36 weeks’ postmenstrual age for oxygen requirement and positive pressure. The primary outcome measured was the change in the incidence of BPD after the introduction of NAVA mode of ventilation. The secondary outcomes measured were supplemental home oxygen therapy at discharge, length of stay, percentage of time spent on the NAVA mode, and invasive ventilator days, compared between the two cohorts. The incidence of BPD in each subgroup was calculated by using the numerator as the number of patients that developed BPD (as defined above) and the denominator being the total number of patients in that subgroup. Length of stay referred to the total number of days spent in hospital, from admission to discharge from the NICU.

Since this was a retrospective study, we could not control the amount of time each neonate spent on the NAVA ventilator. For each neonate in the NAVA group, the percentage of time spent on the NAVA mode of ventilation was calculated for each subgroup. It was calculated as the total number of invasive ventilation hours spent on the NAVA mode as the numerator, divided by the total number of invasive ventilator hours. Based on the percentage of time they spent on NAVA, neonates were divided into five categories: 0%, where neonates spent no time on invasive NAVA (but may have spent time on non-invasive NAVA), 1–24.9%, 25–49.9%, 50–74.9%, and >75% (where neonates spent more than 75% of time on invasive NAVA).

## 3. Statistical Analysis

We performed the descriptive analysis to characterize the study subjects and the distributions of the variables of interest, with the data represented as the mean plus/minus the standard deviation. We examined the changes in patient composition and BPD incidence rates before and after the introduction of the NAVA mode of ventilation. Clinical and demographic information was compared between the control and NAVA cohorts using the two-sample *t*-test, the Wilcoxon rank sum test or the chi-squared test, as appropriate. The association between the introduction of the NAVA mode of ventilation and the risk of BPD incidence was examined using the chi-squared test. A logistic regression analysis was conducted to adjust for the potential confounding effects of other risk factors. We used the chi-squared test and a logistic regression to determine whether BPD incidence was lower in patients receiving the NAVA mode of ventilation, compared to those who did not, after the introduction of NAVA ventilation. Birth weight was tested for normality using the Kolmogorov–Smirnov test (see Supplementary Material Table S1). A *p* value < 0.05 was considered statistically significant.

## 4. Results

Patient characteristics for the study groups are shown in Table 1. Maternal and infant characteristics were not significantly different between the control and NAVA groups, except for the incidence of antenatal steroids. More infants in the NAVA group were exposed to antenatal steroids than in the control group. The mean gestational age was 27.3 ± 2.1 weeks’ in the control group and 26.8 ± 2.2 weeks’ in the NAVA group (*p* = 0.15). There was an almost equal distribution of male and female infants in the two groups.

**Figure 1:**
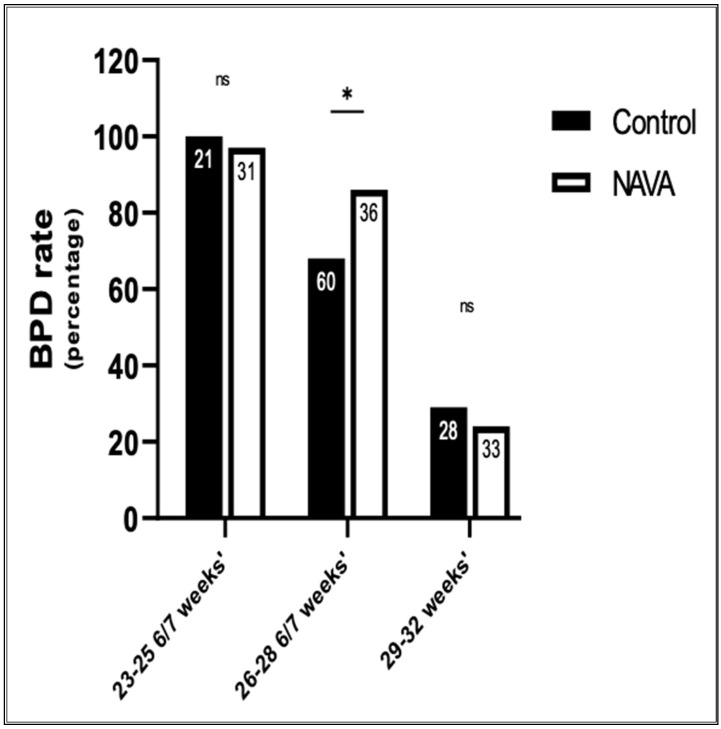
Comparison of BPD rates between the control and NAVA groups. BPD rate is calculated as the total number of patients that developed BPD, as per NICHD guidelines/the total number of patients in that subgroup. The sample size in each subgroup is shown inside each column. * represents significant results, with *p* ≤ 0.5. ns represents non-significant results.

The primary outcome of this study was to compare the incidence of BPD between the control and NAVA groups, as shown in Figure 1. There were no statistical differences in the rates of BPD among the subgroups. In the 23–25 6/7 weeks’ gestation subgroup, 100% (21/21) of infants developed BPD in the control group, compared to 97% (30/31) in the NAVA group (*p* = 0.4). Among the 26–28 6/7 weeks’ gestation subgroup, the control group’s BPD rate was lower, i.e., 68% (41/60), compared to 86% (31/36) in the NAVA group (*p* = 0.05). BPD rates were not different for the NAVA group, compared to the control group, in the 29–32 weeks’ subgroup.

**Figure 2:**
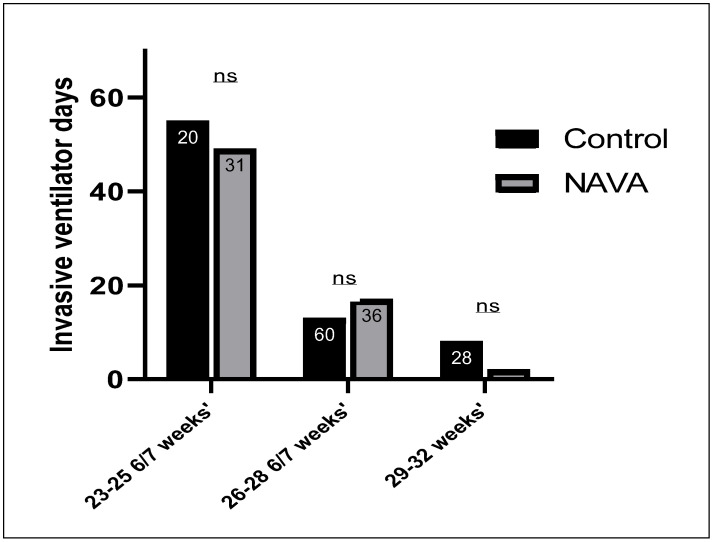
Comparison of the duration of invasive ventilator days among subgroups between the control and NAVA groups. The sample size in each subgroup is shown inside each column. ns represents non-significant results. The NAVA subgroup at 26–28 6/7 weeks’ gestation had more ventilator days than the control group.

Infants in the control group required a longer duration of invasive ventilation, compared to the NAVA group, in the 23–25 6/7 weeks’ gestation subgroup and 29–32 weeks’ gestation subgroup, as shown in Figure 2. Invasive ventilator days were not significantly lower in the control group, compared to the NAVA group, among infants in the 26–28 6/7 weeks’ gestation subgroups.

**Figure 3:**
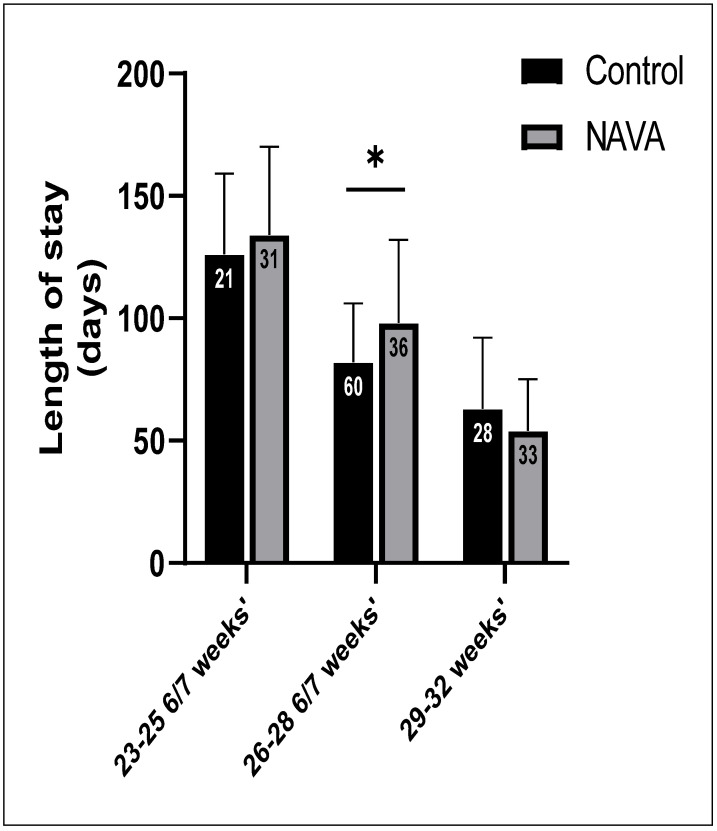
Comparison of length of stay (mean) among subgroups between the control and NAVA groups. The sample size in each subgroup is shown inside each column. Significant results: *p* ≤ 0.5, *.

Length of stay is shown in Figure 3. Infants in the NAVA group had similar lengths of stay to the control group among the 23–25 6/7 weeks’ Gestational age (GA) subgroup (134 ± 36 days vs. 126 ± 33 days, *p* = 0.5). We found that there were longer hospital stays in the NAVA group (98 ± 34 days) compared to the control group neonates (82 ± 24 days) among the 26–28 6/7 weeks’ gestational age subgroup (*p* = 0.01). There was no significant difference in length of stay in the NAVA group compared to the control group in subgroup 29–32 weeks’ gestation (*p* = 0.2).

**Figure 4:**
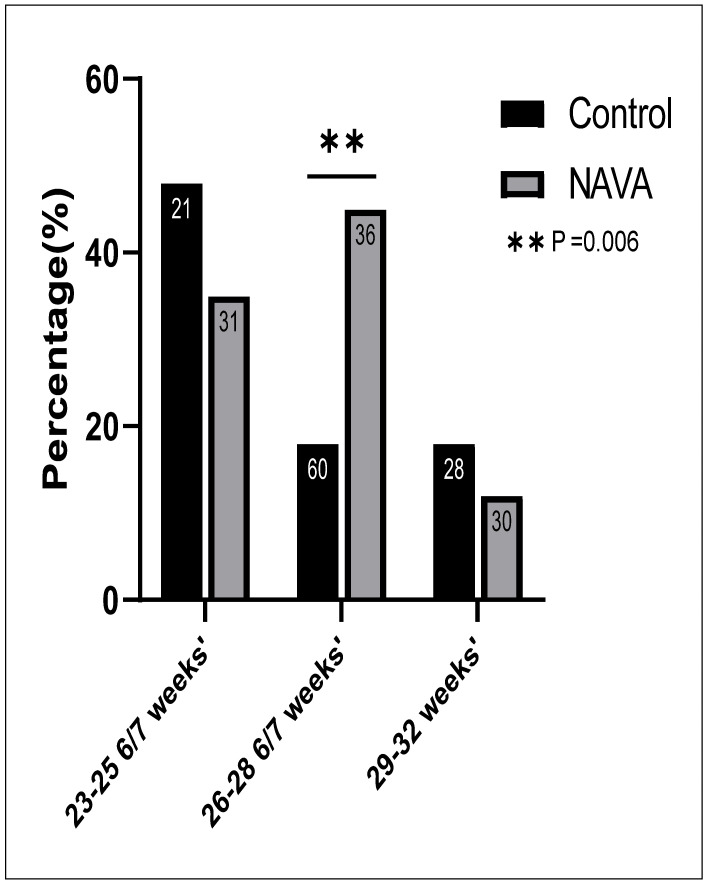
Rates of home oxygen therapy on discharge. Significant results: *p* ≤ 0.01, **. Increased rates of home oxygen therapy in the 26–28 6/7 subgroup who received NAVA.

Depending on the severity of BPD near discharge, neonates were either discharged home on supplemental oxygen, i.e., home oxygen therapy, varying from 1/8 to 1 LPM of 100% oxygen, or they were discharged after tracheostomy and were ventilator-dependent (which was enabled through our home ventilator program). Similar proportions of neonates were discharged on home oxygen in the NAVA group to control group in the 23–25 6/7 weeks’ (35% vs. 48%, *p* = 0.4) and 29–32 weeks’ gestational age (12% vs. 18%, *p* = 0.5) subgroups, as shown in Figure 4. The only significant difference was observed in the 26–28 6/7 weeks’ gestation subgroup, with a surprisingly high number of patients discharged home on oxygen in the NAVA group compared to controls (45% vs. 18%, respectively, *p* = 0.006).

In the 26–28 6/7 weeks’ subgroup, a significantly higher number of patients needed a tracheostomy and were ventilator-dependent in the NAVA group than in the control group (3/36 vs. 0/60, *p* = 0.02).

Table 2 shows the results of a multistep logistic regression comparing the relationships between the primary outcome of BPD and other potential confounders. In the initial model, multiple confounders were added, including gestational age, preterm labor, post-natal steroids, Vitamin A, caffeine, infection, NEC (stage 2 and above), intraventricular hemorrhage, and patent ductus arteriosus (PDA). Only the variables shown in Table 2 were found to be relevant. Gestational age was significantly associated with BPD in our study, with an odds ratio of 27. Additionally, the data showed that caffeine therapy reduced the risk of BPD.

**Figure 5:**
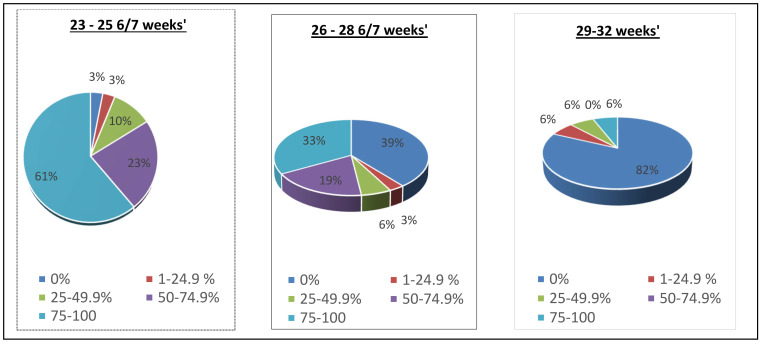
Pie chart representation of the percentage of time each neonate spent on invasive NAVA in consecutive subgroups and then grouped into five categories as 0% time to 75–100% time on invasive NAVA.

Subgroups were also compared, to evaluate the mean percentage of time (hours) spent on the NAVA mode of ventilation, compared to the total number of intubated hours of each subgroup in the NAVA group, as shown in Figure 5. Neonates in the subgroup 23–25 6/7 weeks’ GA spent 84% of their intubated time on the invasive NAVA mode of ventilation; the subgroup 26–28 6/7 weeks’ GA spent 52% of their intubated time on invasive NAVA; and the 29–32 weeks’ GA subgroup spent 6% of their intubated time on invasive NAVA. For the intubated time not spent on NAVA, synchronized intermittent mandatory ventilation pressure control was the second most common mode, followed by high frequency jet and oscillatory ventilation, across all gestational age subgroups.

## 5. Discussion

BPD is a multifactorial disease process involving an imbalance between pro- and anti-inflammatory factors, hereditary factors, prematurity, oxidant injury, and ventilator-induced lung injury. Patient-triggered ventilation has been shown to improve alveolar ventilation, oxygenation, and cardiovascular stability and to reduce stress (as measured by epinephrine levels). However, only non-invasive respiratory support and volume targeted ventilation have been shown to reduce the risk of BPD. NAVA allows neonates to control the frequency, timing, and magnitude of lung inflation, which decreases asynchrony, possibly leading to decreased incidences of lung trauma and inflammation.

This study evaluated the effects of introducing the NAVA mode of ventilation on BPD outcome. We found that using NAVA in conjunction with other modes of ventilation does not decrease the incidence of BPD, irrespective of gestational age. We found no difference in rates of BPD overall, but there was an unexpected finding of a higher incidence of BPD among the 26–28 6/7 weeks’ gestation subgroup associated with NAVA. This could be due to the unequal sample size of the subgroup (*n* = 60 vs. 36) in the control and NAVA groups, respectively as well as a significantly higher number of females in the control group than in the NAVA group (53% vs. 30% *p* = 0.03), which could have skewed the data in favor of the control group. Significant differences among sex and sample size were not observed in other subgroups.

Another reason we may not have found any difference among the subgroups could be due to the possibility of neonates spending more time in back-up settings on the NAVA ventilator, which is a pressure control mode (Synchronized Intermittent Mandatory ventilation- pressure control, SIMV-PC). Since this is a retrospective study, the percentage of time spent in the back-up pressure control mode in the NAVA group could not be determined, as such data are not recorded routinely. Lee et al. [33], Kallio et al. [34], and Di Mussi et al. [35] showed that NAVA significantly decreased the peak pressures and work of breathing, compared to SIMV + PS or PSV, in preterm infants. But none of these articles looked at the long term outcomes, such as changes in the incidence of BPD. One can suggest that, by decreasing peak inspiratory pressures using the NAVA mode, there would also be less biotrauma, less inflammation and a possible decrease in the risk of BPD. But our study did not show such benefit.

As this was a retrospective study, NAVA ventilation was not always used at the beginning of the patients’ clinical course and sometimes was used when BPD was already established. This may have been another reason for not finding a difference in the incidence of BPD. NAVA mode effectiveness comes from lowering Peak inspiratory pressure (PIP) and decreasing the duration of invasive ventilation [36], but it is possible that our patients were on the NAVA mode late in their clinical course of the disease process, i.e., BPD. The postnatal age at which patients were put on NAVA was not collected.

We were very surprised to find a significantly higher number of neonates in the 26–28 6/7 weeks’ gestation subgroup being discharged home on supplemental oxygen, as well as a higher number of patients who received a tracheostomy, in the NAVA group than in the control group. Various clinical predictors of home oxygen therapy have been reported previously, such as gestational age, duration of mechanical ventilation, PDA, and the use of antenatal steroids [37,38]. There were no significant differences in these predictors in the 26–28 6/7 weeks’ gestational age subgroup.

The length of stay in the NICU is a more complicated variable, as it can be affected by various prematurity-related conditions, rather than the duration and mode of ventilation alone. Therefore, any difference in the length of hospital stay cannot be attributed to the introduction of a new mode of ventilation. In our study, we found that neonates in the 26–28 6/7 weeks’ gestational age subgroup had significantly longer lengths of stay in the NAVA group, compared to the control group. Overall, the length of stay across all subgroups were comparable to other studies [39]. A randomized controlled trial in 60 preterm infants between 28 weeks’ and 36 + 6 weeks’ gestation compared the NAVA mode with conventional pressure-limited, time-cycled ventilation in the management of respiratory distress syndrome (RDS) [34]. They found no significant differences between outcomes such as duration of invasive ventilation and hospital days. Similar trends were found when NAVA was used in other diseases and older infants. Piastra et al. [40] compared pediatric patients with Acute respiratory distress Syndrome (ARDS) on pressure support ventilation vs. NAVA, and found no difference in the length of stay, despite improvements in vital and physiological parameters, along with a decrease in the duration of mechanical ventilation. A randomized control trial [41] examined 175 pediatric patients post-operatively on NAVA vs. conventional ventilation. They also found no differences for the median number of ventilator days of the length of stay.

## 6. Limitations

This is a single center, retrospective cohort study with a limited number of patients in each subgroup, especially in the NAVA 26–28 6/7 weeks’ gestation subgroup. The NAVA group in our study was nonexclusive, being used in conjunction with other modes of ventilation, so any difference in outcomes cannot be entirely due to NAVA use. We cannot find out, retrospectively, how much time the patients spent in the back-up pressure-controlled mode in NAVA. Since NAVA was adopted by our unit in 2015, there was a “NAVA learning curve” among the group. Patients could have already developed or were in the process of developing BPD by the time they were placed on the NAVA mode of ventilation. There had been changes in clinical practice, such as the introduction of feeding protocols with an emphasis on early fortification of enteral feeds, early aggressive parenteral nutrition, and the revision of ventilation guidelines in the last year of the study period. All these factors could have influenced respiratory outcomes.

## 7. Conclusions

In summary, we found that the NAVA mode of ventilation was not associated with a reduction in the incidence of BPD, when compared to other modes of ventilation. There were some unexpected increases in length of stay, tracheostomy rates, and home oxygen therapy rates in one of the NAVA subgroups. Although this study showed no difference in BPD, the use of NAVA may not have been early enough in the clinical course. Large randomized controlled trials comparing NAVA with volume-targeted ventilation are clearly needed to answer this question.

## Figures and Tables

**Table 1:** Baseline characteristics of infants in the control and NAVA groups.

Characteristic	Control (*n* = 109)	NAVA (*n* = 97)	*p* Value
1. Gestational age in weeks (mean ± SD)	27.3 ± 2.1	26.8 ± 2.2	0.15
2. Sex			
Males (%)	54%	56%	0.82
Females (%)	46%	44%	
3. Birth weight in grams (mean ± SD)	1007 ± 300	955 ± 298	0.21
4. Antenatal steroids (%)	80%	89%	0.04
5. APGAR 1 min (median)	4	5	0.2
6. APGAR 5 min (median)	7	7	0.18
7. Chorioamnionitis (%)	6%	3%	0.4
8. Preterm labor (%)	42%	54%	0.1
9. Premature rupture of membranes (%)	6%	12%	0.08
10. Preeclampsia (%)	25%	24%	0.9
11. Placental abruption (%)	8%	12%	0.3

**Table 2:** Logistic regression between BPD (primary outcome) and confounder variables. * represents significant results (*p* < 0.01).

Variable	Odds Ratio	95% Confidence Interval	*p* Value
Gestational age	27	2.3–307.3	0.0013 *
Infection	4	0.98–13.8	0.05
Vitamin A	0.9	0.28–2.7	0.8
Caffeine	0.3	0.08–0.98	0.05
PDA	1	0.28–3.64	0.9

## Data Availability

The data presented in this study are available on request from the corresponding author. The data are not publicly available due to data contains information that could compromise the privacy of research participants.

## Supplementary Materials

The following supporting information can be downloaded at: https://www.mdpi.com/article/10.3390/children11010113/s1, Table S1: Test for normality using the Kolmogorov–Smirnov test.
