# Supplementary material for: Trends in the Incidence of Bronchopulmonary Dysplasia after the Introduction of Neurally Adjusted Ventilatory Assist (NAVA)
Source: Children (Basel). 2024 Jan 17;11(1):113. doi: 10.3390/children11010113 (PMC10814022; doi:10.3390/children11010113)
Supplement: Supplementary file 1 [file children-11-00113-s001.zip › children-2589284-supplementary.pdf]

Supplementary material

Kolmogorov-smirnov test was done for birth weight to test if groups had identical distribution among Control and NAVA groups using SPSS software (29.0.2.0) since the observed sample size is greater that 50 (n>50 in each group). The birth weight of D (97) for control group and NAVA group with P values of 0.2 and 0.066 respectively (Table S1), do not indicate deviation from normality.

Table S1: Test for Normality using Kolmogorov-smirnov test

	Statistics	df	Sig. (P<0.05 =significant)
Control	0.072	97	0.200
NAVA	0.087	97	0.066
